# The gender gap in mobility: A global cross-sectional study

**DOI:** 10.1186/1471-2458-12-598

**Published:** 2012-08-02

**Authors:** Samia Djemâa Mechakra-Tahiri, Ellen E Freeman, Slim Haddad, Elodie Samson, Maria Victoria Zunzunegui

**Affiliations:** 1 Centre de Research de Université de Montréal, Montreal, Quebec, Canada; 2 Department of Ophthalmology, Université de Montréal, Montreal, Quebec, Canada; 3 Centre de Recherche de Hôpital Maisonneuve-Rosemont, Montreal, Quebec, Canada

**Keywords:** Gender, Inequality, Mobility, Human development index, Gender-related development index

## Abstract

**Background:**

Several studies have demonstrated that women have greater mobility disability than men. The goals of this research were**:** 1) to assess the gender gap in mobility difficulty in 70 countries; 2) to determine whether the gender gap is explained by sociodemographic and health factors; 3) to determine whether the gender gap differs across 6 regions of the world with different degrees of gender equality according to United Nations data.

**Methods:**

Population-based data were used from the World Health Survey (WHS) conducted in 70 countries throughout the world. 276,647 adults aged 18 years and over were recruited from 6 world regions. Mobility was measured by asking the level of difficulty people had moving around in the last 30 days and then creating a dichotomous measure (no difficulty, difficulty). The human development index and the gender-related development index for each country were obtained from the United Nations Development Program website. Poisson regression with Taylor series linearized variance estimation was used.

**Results:**

Women were more likely than men to report mobility difficulty (38% versus 27%, P < 0.0001). The age-adjusted prevalence rate ratio for female gender was 1.35 (95% CI 1.31–1.38). The addition of education, marital status, and urban versus rural setting reduced the prevalence rate ratio to 1.30 (95% CI 1.26–1.33). The addition of the presence of back pain, arthritis, angina, depressive symptoms, and cognitive difficulties further reduced the prevalence rate ratio to 1.12 (95% CI 1.09–1.15). There was statistical interaction on the multiplicative scale between female gender and region (P < 0.01). The Eastern Mediterranean region, which had the greatest loss of human development due to gender inequality, showed the largest gender gap in mobility difficulty, while the Western Pacific region, with the smallest loss of human development due to gender inequality, had the smallest gender gap in mobility difficulty.

**Conclusions:**

These are the first world-wide data to examine the gender gap in mobility. Differences in chronic diseases are the main reasons for this gender gap. The gender gap seems to be greater in regions with the largest loss of human development due to gender inequality.

## Background

Mobility loss is an important global public health issue because it often represents a pre-clinical stage of disability and because it is associated with severe disability, death, and large healthcare expenditures [1-4]. Many studies have demonstrated that women have greater mobility disability than men [5-9]. This gap is thought to be due to a greater incidence of mobility disability in women rather than differences in recovery from disability or mortality [6]. Reasons for the gap in mobility disability are not entirely clear but are at least partly due to a greater risk of certain diseases in women (arthritis, depression) that are important to mobility [10-12]. Life course exposures such as physical activity, smoking, diet, childhood hunger, poverty, and body mass index have also been investigated to explain the gender gap. However, two studies did not find evidence that these factors explained the gender gap in mobility [13] and concluded that additional biological and social factors were needed to explain differences in mobility between men and women [14]. Interestingly, there is evidence from a population-based study in Sweden that the gender gap in mobility disability has diminished over time, perhaps as gender equality has improved. In Sweden, the age-adjusted odds ratios for having difficulty climbing stairs, running 100 meters, and walking 100 meters in women versus men decreased from 1968 to 1991 [15]. For example, the age-adjusted odds ratio for having difficulty walking up stairs in women was 2.1 in 1968 while it dropped to 1.5 in subsequent years. This observation led us to question whether the gender gap is different in world regions with different levels of gender equality. The gender mobility gap may be the result of latent and cumulative differences in exposure to mobility risk factors from birth to old age.

The World Health Survey (WHS) data, in which population-based data were collected from 70 countries throughout the world, provide a unique opportunity to examine the gender gap in mobility disability in regions with different profiles of gender equality. We hypothesized that regions with more gender inequality would have a greater difference in mobility disability between women and men. We have recently documented the gender mobility gap in three West African countries using data from WHS [16]. Here, we analysed WHS data to 1) assess the gender gap in mobility difficulty in 70 countries of the world; 2) to determine whether the gender gap is explained by sociodemographic and health factors; 3) to determine whether the gender gap differs across 6 regions of the world with different degrees of gender equality.

## Methods

### Study design and population

The World Health Survey (WHS) is a large, population-based, cross-sectional study that was conducted in 2002–2003 by the World Health Organization (WHO) in 70 countries including 30 European, 18 African, 7 North and South American, 4 Eastern Mediterranean, 5 Southeast Asian, and 6 Western Pacific countries. Detailed information about the methods of the World Health Survey is available online [17]. A multi-stage, stratified, random cluster sampling strategy was used to identify the participants to be contacted in each country. All sampling plans were reviewed by WHO before implementation. Sampling strata were created based on 3 factors: region, socioeconomic status, and presence of a healthcare facility. Lists of households were obtained from population registries, voter lists, manual enumeration, or other methods. Households were randomly sampled in the selected areas, and within each household, an adult who was 18 years or older was selected using a Kish table. Non-response was carefully documented. Response rates were very good with an average household response rate of 87% and an average individual response rate of 97%. Informed consent was obtained from all participants and ethics approval was obtained by local institutional review committees. The research complied with the tenets of the Declaration of Helsinki. Also, the Comité d’éthique de la recherche at Maisonneuve-Rosemont Hospital in Montreal approved our use of these data.

### Data collection

Participants answered a face-to-face interviewer-administered questionnaire that was translated into 68 local languages and back-translated using a standard WHO protocol [18]. Briefly, forward translation was done locally by a bilingual multidisciplinary group. Back-translation was then performed by an independent group and was reviewed at the WHO. Any discrepancies were resolved. Finally, a review of the translated questionnaire was performed by a panel of experts.

Mobility difficulty was measured by asking: “Overall in the last 30 days, how much difficulty did you have with moving around?”. Responses were rated on a scale of 1 to 5 as follows: 1: None; 2: Mild; 3: Moderate; 4: Severe; 5: Extreme/Cannot do. A dichotomous variable was then created such that those who reported “mild, moderate, severe, or extreme difficulty/cannot do” were compared to those who reported no difficulty. Questions were asked about age, marital status, and level of formal education. The interviewer observed whether the participant lived in an urban, semi-urban, or rural area.

Participants were asked if they had ever been diagnosed with angina or arthritis and if they had experienced the following in the last 30 days : back pain (yes or no), difficulty remembering or concentrating on things (none, mild, moderate, severe, extreme/cannot do). In addition, participants were asked if they had experienced a period of several days when they felt sad, empty, or depressed over the last 12 months (yes, no).

### Country-level data

**Figure 1:**
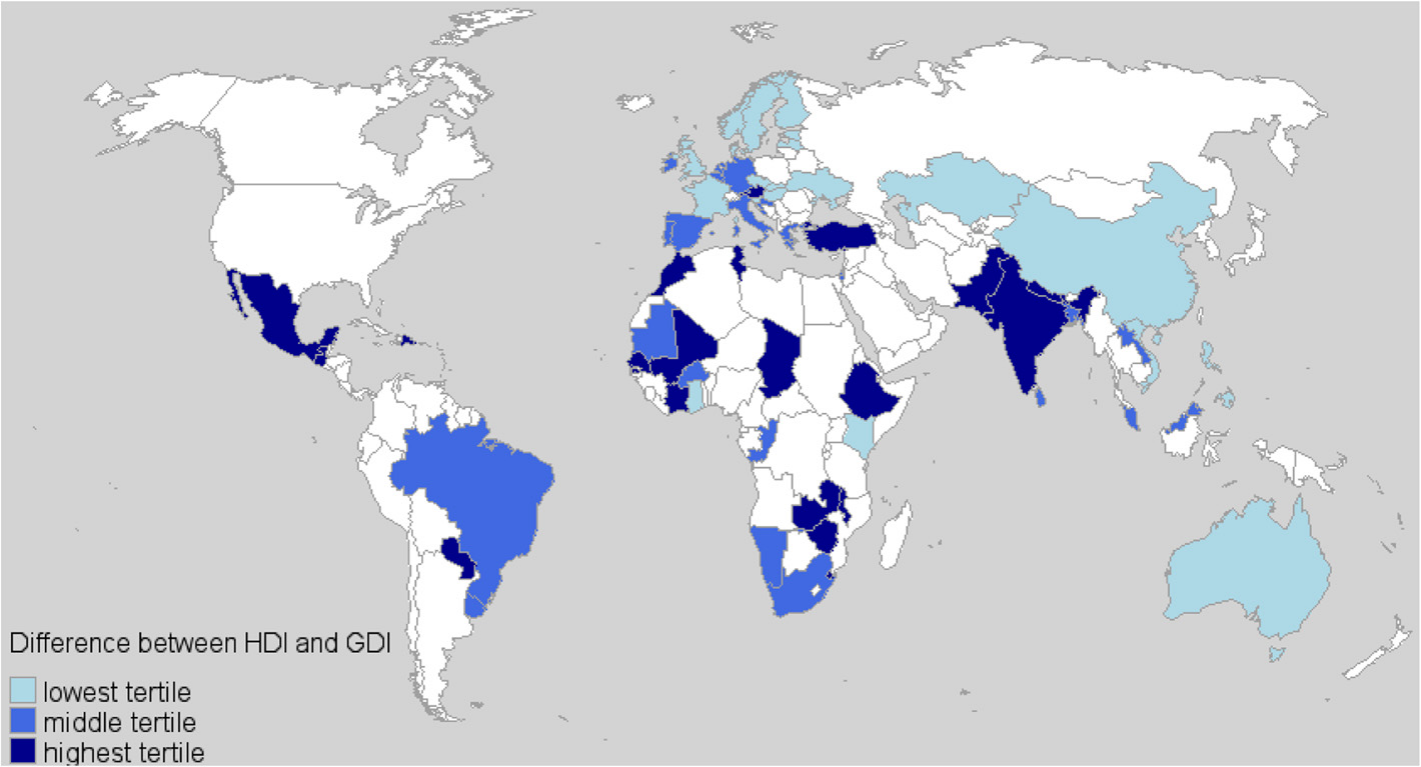
** WHS countries by difference between scores on the Human Development and Gender Development Indices**.

Country-level data was added to the WHS dataset. In order to determine the level of gender inequality in a country, data from 2003 were obtained on two variables from the United Nations Human Development website [19]. The human development index (HDI) is a composite of 3 factors: life expectancy, educational attainment, and income. The gender-related development index (GDI) measures the same factors as the HDI but the index is penalized for the size of the gap between men and women. The GDI is not meant to be analyzed by itself but rather as a difference or a ratio with the HDI. Bigger differences indicate greater loss of human development due to gender inequality [20]. Table 1 presents all the countries in the WHS and Figure 1 is a map indicating whether the countries are in the lowest tertile of the HDI-GDI difference, the middle tertile, or the highest tertile.

**Table 1:** Countries that participated in the WHS

***Region***	***Countries***
Africa	Burkina Faso, Chad, Côte d'Ivoire, Congo, Comoros, Ethiopia, Ghana, Kenya, Mali, Mauritania, Malawi, Mauritius, Namibia, Senegal, Swaziland, South Africa, Zambia, Zimbabwe
Americas	Brazil, Dominican Republic, Ecuador, Guatemala, Mexico, Paraguay, Uruguay
Eastern Mediterranean	Morocco, Pakistan, Tunisia, United Arab Emirates
Europe	Austria, Belgium, Bosnia and Herzegovina, Croatia, Czech Republic, Denmark, Estonia, Finland, France, Georgia, Germany, Greece, Hungary, Ireland, Israel, Italy, Kazakhstan, Latvia, Luxembourg, Netherlands, Norway, Portugal, Russian Federation, Slovakia, Slovenia, Spain, Sweden, Turkey, Ukraine, United Kingdom
Southeast Asia	Bangladesh, India, Sri Lanka, Myanmar, Nepal
Western Pacific	Australia, China, Lao People's Democratic Republic, Malaysia, Philippines, Viet Nam

### Statistical analysis

Demographic and health characteristics were compared between men and women and between those with and without mobility difficulty. Percentages and means were adjusted for the complex survey design. Three countries (Slovenia, Guatamala, Zambia) did not report survey design information and were therefore excluded from all analyses. Differences were tested using Pearson’s chi-square tests or Student’s t-tests taking the complex survey design into account. A series of Poisson regression models with Taylor series linearized variance estimation were performed to examine 1) whether there was a gender gap in mobility difficulty; 2) what sociodemographic or health variables primarily explained the gender gap; and 3) whether the gender gap differed across the 6 world regions (i.e. whether there was interaction between gender and region). We adjusted for the following variables based on their availability in the dataset and prior research indicating their importance to mobility: age, marital status, education, rural versus urban setting, arthritis, angina, depression, back pain, and cognitive difficulty [9,11,21]. Interaction was examined by stratification. It was then statistically assessed on a multiplicative scale by creating interaction terms between gender and each region with Africa arbitrarily being chosen as the reference region given it was the region listed first alphabetically. Regression analyses took into account the complex survey design. All analyses were done using the survey estimation (SVY) commands in STATA/IC software version 11.2 (College Station, Texas, USA).

## Results

Table 2 shows the distribution of characteristics of men and women in the WHS. There were statistically significant differences between men and women for all sociodemographic and health variables. The mean ages for men and women in the WHS were 38 and 40 years old respectively. Men were more likely to never have married than women while women were more likely to be widowed (P < 0.01). Women were more likely to not have any formal education and to report all chronic conditions (P < 0.01).

**Table 2:** Characteristics of men and women in WHS (n = 256,286)

***Characteristic***	***Men (n = 126,195)***	***Women (n = 130,091)***	***P values***
	***% or Mean (SD)***	***% or Mean (SD)***	
**Age, years**	38.4 ± 16.6	39.7 ± 16.7	0.029
**Marital Status**			
Married/cohabiting	66.2	65.4	<0.0001
Never married	29.1	18.0	
Separated/divorced	2.4	5.1	
Widowed	2.3	11.4	
**Education**			
No school	18.8	30.0	<0.0001
Less than primary	10.9	10.7	
Primary completed	20.6	17.7	
Secondary completed	21.5	18.1	
Greater than secondary	28.2	23.6	
**Setting**			
Urban	45.6	48.1	<0.0001
Rural	54.5	52.0	
**Back pain**			
Yes	28.8	40.4	<0.0001
No	71.2	59.6	
**Arthritis**			
Yes	10.1	15.7	<0.0001
No	89.9	84.3	
**Angina**			
Yes	5.7	8.0	<0.0001
No	94.3	92.0	
**Depression**			
Yes	23.4	33.1	<0.0001
No	76.6	66.9	
**Cognitive Difficulties**			
Any	30.5	42.7	<0.0001
None	69.5	57.3	

In Table 3, we examined the characteristics of those with and without mobility difficulty. Women were more likely than men to report mobility difficulty (38% versus 27%, P < 0.01). Those who were older, had less formal education, lived in rural areas, and had chronic health conditions were more likely to report mobility difficulty (P < 0.01). Marital status was also associated with mobility difficulty such that those who never married had the least mobility difficulty (P < 0.01).

**Table 3:** Characteristics of the study population with and without mobility difficulty

	***Mobility Difficulty***	***No Mobility Difficulty***	***P values***
	***% or Mean ± SD***	***% or Mean ± SD***	
**Gender**	27.1	72.9	<0.0001
Men	37.9	62.1	
Women			
**Age**	46.2 ± 18.3	35.6 ± 14.7	<0.0001
**Marital Status**	18.6	81.4	<0.0001
Never married	34.3	65.7	
Married/cohabiting Separated/divorced Widowed			
	32.1	67.9	
	63.1	37.0	
**Education**	45.0	55.0	<0.0001
No school	39.0	61.0	
Less than primary	32.4	67.6	
Primary completed	27.0	73.0	
Secondary completed	22.2	77.8	
Greater than secondary			
**Setting**	28.5	71.5	<0.0001
Urban	36.4	63.6	
Rural			
**Back pain**	49.5	50.5	<0.0001
Yes	23.8	76.2	
No			
**Arthritis**	61.2	38.8	<0.0001
Yes	28.1	71.9	
No			
**Angina**	62.5	37.5	<0.0001
Yes	30.0	70.0	
No			
**Depression**	46.7	53.3	<0.0001
Yes	26.6	73.4	
No			
**Cognitive difficulties**	56.9	43.1	<0.0001
Any	18.5	81.5	
None			

In Table 4, the prevalence rate ratios for female gender are given adjusting for different sets of covariates while holding the number of observations constant. The age-adjusted prevalence rate ratio for gender in Model 1 was 1.35 (95% CI 1.31-1.38). In other words, women had a 35% higher prevalence of mobility difficulty than men. The addition of education, marital status, and urban versus rural setting in Model 2 slightly reduced the prevalence rate ratio for female gender to 1.30 (95% CI 1.26-1.33). Adding the 5 health variables (back pain, arthritis, angina, depressive symptoms, and cognitive difficulties) in Model 3 further reduced the prevalence rate ratio for female gender to 1.12 (95% CI 1.09-1.15).

**Table 4:** Poisson regression models showing the changes in the gender gap in mobility difficulty after adjustment

	***Model 1 (n = 212,744)***	***Model 2 (n = 212,744)***	***Model 3 (n = 212,744)***
	***Mobility Difficulty***	***Mobility Difficulty***	***Mobility Difficulty***
	***PRR (95% CI)***	***PRR (95% CI)***	***PRR (95% CI)***
**Gender**			
Men	1.00	1.00	1.00
Women	1.35 (1.31–1.38)	1.30 (1.26–1.33)	1.12 (1.09–1.15)
**Age**	1.02 (1.02–1.02)	1.02 (1.02–1.02)	1.01 (1.01–1.01)
**Marital Status**			
Married or cohabiting		1.00	1.00
Never married		0.86 (0.82 –0.91)	0.87 (0.83 –0.92)
Separated/Divorced		0.93 (0.87 –0.99)	0.88 (0.78 –1.02)
Widowed		1.04 (1.00 –1.08)	0.99 (0.96 –1.02)
**Education**			
Greater than secondary		1.00	1.00
Secondary completed		1.17 (1.11 –1.23)	1.15 (1.10 –1.20)
Primary completed		1.29 (1.22 –1.36)	1.19 (1.14 –1.25)
Less than primary		1.40 (1.32 –1.47)	1.22 (1.16 –1.28)
No school		1.48 (1.41 –1.56)	1.28 (1.22 –1.34)
**Setting**			
Rural vs. Urban		1.20 (1.16 –1.25)	1.21 (1.17 –1.26)
**Back pain**			
Yes vs. No			1.35 (1.31 –1.39)
**Arthritis**			
Yes vs. No			1.25 (1.22 –1.28)
**Angina**			
Yes vs No			1.19 (1.15 –1.22)
**Depression**			
Yes vs. No			1.19 (1.16, 1.22)
**Cognitive Difficultie**s			
Any vs. None			2.23 (2.16 – 2.30)

PRR = prevalence rate ratio.

We then examined whether the gender gap in mobility difficulty differed across world regions by examining region-specific regression models (Table 5). After age adjustment, the gender gap was the largest in the Eastern Mediterranean (prevalence rate ratio (PRR) = 1.66, 95% CI 1.51-1.81) and was the smallest in the Western Pacific (PRR = 1.12, 95% CI 1.06-1.18). In the fully adjusted model (Model 3), that pattern remained with the gender gap in the Western Pacific completely disappearing (PRR = 1.03, 95% CI 0.98-1.08). Adding interaction terms for gender and world region to an age-adjusted model for all regions combined resulted in statistically significant interactions for the Western Pacific and the Eastern Mediterranean regions compared to Africa (data not shown in Table 5, P < 0.001 and P < 0.001 respectively). In other words, the prevalence rate ratio for female gender in the Eastern Mediterranean was significantly larger than in Africa while the prevalence rate ratio in the Western Pacific was significantly smaller than in Africa. Consistent with this result, of the 6 world regions in the WHS, countries in the Western Pacific region had the smallest difference between the HDI and the GDI while countries in the Eastern Mediterranean region had the largest.

**Table 5:** Poisson regression models showing the changes in the gender gap in mobility difficulty by world region

**World Region**	**% with**	**Model 1 †**	**Model 2 ‡**	**Model 3 §**
	**Mobility Difficulty in**	**Mobility Difficulty**	**Mobility Difficulty**	**Mobility Difficulty**
	**Men and Women**	PRR* (95% CI)	PRR* (95% CI)	PRR* (95% CI)
**Africa, n = 60,277**	23%, 31%	1.30 (1.23–1.37)	1.26 (1.20 –1.34)	1.11 (1.06 –1.17)
**Americas, n = 49,803**	17%, 25%	1.37 (1.26 –1.49)	1.38 (1.26–1.50)	1.16 (1.06 –1.26)
**Eastern Mediterranean n = 16,450**	24%, 40%	1.66 (1.51 –1.81) ||	1.49 (1.36 –1.62)	1.17 (1.07 –1.27)
**Europe n = 40,477**	25%, 38%	1.34 (1.24 –1.44)	1.32 (1.23 –1.42)	1.11 (1.04 –1.19)
**South-east Asia, n = 34,743**	35%, 49%	1.39 (1.33 –1.45)	1.30 (1.24 –1.36)	1.16 (1.12 –1.21)
**Western Pacific, n = 27,236**	31%, 36%	1.12 (1.06 –1.18) ||	1.09 (1.03 –1.15)	1.03 (0.98 –1.08)

* Prevalence rate ratio for women versus men.

**†** Model 1: Adjusted by age.

**‡** Model 2: Adjusted by age, marital status, education, and setting.

**§** Model 3: Adjusted by age, marital status, education, setting, back pain, arthritis, angina, depression, and cognitive difficulties.

|| In a single regression model for all regions combined (not shown here), interaction terms were statistically significant for the Eastern Mediterranean and the Western Pacific regions using Africa as the reference region (P < 0.01).

The countries listed as part of the Eastern Mediterranean region by the WHS included Morocco, Pakistan, Tunisia, and the United Arab Emirates (UAE). These countries differ substantially in economic development, religious freedom, and geography. They share in common large Muslim populations. Despite the diversity of these 4 countries, there was not substantial heterogeneity in the age-adjusted prevalence rate ratios for female gender in each of these 4 countries as the prevalence rate ratios ranged from 1.54 (95% CI 1.12, 2.13) in the United Arab Emirates to 1.96 (95% CI 1.72, 2.23) in Tunisia. Furthermore, the differences between the human development index and the gender development index for 4 of the 5 countries were in the largest quartile of all the WHS countries (The UAE did not have data on the gender development index). At the other end of the gender equality spectrum, the countries listed as part of the Western Pacific by the WHS included Australia, China, Malaysia, Philippines, Lao People’s Democratic Republic, and Vietnam. Again, despite the diversity of these countries, the age-adjusted prevalence rate ratios for female gender were fairly similar ranging from 1.04 (95% CI 0.99, 1.10) in the Philippines to 1.39 (95% CI 1.15, 1.68) in China. The differences between the human development index and the gender development index for 4 of the 6 countries were in the smallest quartile of all the WHS counties while Malaysia and Lao were in the smallest 30^th^ percentile.

## Discussion

This is the first study to use world-wide data to examine the gender gap in mobility disability. The WHS included data from 70 high, middle, and low income countries providing a unique opportunity to compare the gender gap in these diverse socioeconomic and cultural environments. We found support for our hypothesis that regions with more gender inequality would have a greater difference in mobility difficulty between women and men. The region with the largest gender gap in mobility difficulty was the Eastern Mediterranean, which also had the greatest difference between HDI and GDI indicating greater loss of human development due to gender inequality. Conversely, the region with the smallest gender gap in mobility difficulty was the Western Pacific, which had the smallest difference between the HDI and GDI among the 6 world regions of the WHS.

The risk of mobility disability can be reduced by minimizing the risk of chronic conditions by following preventive health practices like getting enough physical activity, treating hypertension and high cholesterol, eating a healthy diet, maintaining a healthy body weight, and not smoking [22]. Clearly, the ability to follow these preventive health practices is impaired without sufficient autonomy and resources, a reality for many men and even more women in the world today. The opportunity to live a healthy lifestyle is essential and it may be deprived of women living in areas with great gender inequality.

Most prior research on the gender gap in mobility disability has been done in the United States [5,6,8,9]. A few studies have been done in middle or low income countries. For example, data from a survey of older adults living in 6 Latin American and Caribbean cities found that women were more likely than men to have lower extremity limitations (OR = 2.29, 95% CI 2.04, 2.79) [23]. A study of 604 older adults in rural Guatemala found that women were more likely than men to report gross mobility disability after age adjustment (OR = 2.10, P < 0.001) [21]. Women had greater disability in activities of daily living than men in an older, urban Brazilian population (OR = 2.16, 95% CI 1.32, 3.55) that persisted even after adjustment for social and health conditions [24]. Two additional studies in the Eastern Mediterranean region have previously reported the gender mobility gap [25,26]. Our results greatly add to this prior research by examining this issue on a world-wide basis and including all adults over 18 years of age rather than just older adults.

We found that the gender gap was attenuated after adjusting for health factors. Previous studies done in older adults have also reported this, emphasizing arthritis and obesity as two major reasons for the gender gap [9]. Another reason that could explain the gender gap in mobility disability includes an increased risk of sarcopenia in older women [27]. In our data, which included adults of all ages, the two health conditions that caused the biggest decreases in the prevalence rate ratio for gender were back pain and cognitive difficulties. Sociodemographic factors besides gender that were associated with mobility difficulty included older age, being widowed, having no formal education, and living in a rural setting (P < 0.05). A report of a diagnosis of arthritis, a diagnosis of angina, and report of difficulty with cognitive tasks like remembering things or concentrating were also associated with mobility difficulty (P < 0.05).

The major strength of this study is that it includes population-based data from 70 countries from diverse economic and cultural backgrounds. We added to this dataset by including country-level data on gender inequality from the United Nations database. The large sample size in the WHS allowed us to test whether the gender gap in mobility difficulty differed by region, which had never been done before. A limitation of this dataset is that the data are collected by self-report. However, the question on mobility difficulty used by the WHS correlates well with the Timed Up and Go test, an objective measure of mobility [28]. In a sample of 161 older adults at Hôpital Maisonneuve-Rosemont, we found a moderate correlation between the WHS question on mobility difficulty and the Timed Up and Go time in which people are asked to rise from a seated position, walk 3 meters across the room, and return to the chair (r = 0.51, P < 0.01). In addition, as proof of construct validity, we have shown that answers to this question correlate strongly with mobility risk factors in this paper and in our previous publication in 3 African countries [16]. Another limitation is that the cross-sectional nature of this dataset does not allow us to examine the onset or duration of mobility difficulty.

## Conclusions

Women reported greater mobility difficulty than men and this difference was attenuated after adjustment for chronic health conditions. Furthermore, our data suggest that the gender gap in mobility difficulty is greatest in regions that are losing the most human development due to gender inequality. More attention must be devoted to empowering and encouraging men and women across the world to follow preventive health practices like getting enough physical activity, treating hypertension and high cholesterol, eating a healthy diet, maintaining a healthy body weight, and not smoking [22].

## Competing interests

The author(s) declare that they have no competing interests.

## Authors’ contributions

ST added the country-level data to the dataset, did analyses, and helped to write the manuscript; EEF did analyses, supervised the project, and helped to write the manuscript; SH provided expertise on the analysis and manuscript; ES cleaned the WHS data, constructed the world-wide WHS dataset, and reviewed the manuscript; MVZ provided expertise on the analysis and manuscript and provided funding for the project. All authors read and approved the final manuscript.

## Pre-publication history

The pre-publication history for this paper can be accessed here:

http://www.biomedcentral.com/1471-2458/12/598/prepub
